# The impact of “long COVID” on menstruation in Chinese female college students and the intervention of acupuncture

**DOI:** 10.1097/MD.0000000000036818

**Published:** 2024-02-09

**Authors:** Juwei Dong, Jinxia Ni, Ziniu Zhang, Haoyue Yan, Jingni Xu, Jingjing Zhao

**Affiliations:** a Department of Acupuncture and Moxibustion, Beijing University of Chinese Medicine Affiliated Dongzhimen Hospital, Beijing, China.

**Keywords:** acupuncture, COVID-19, female college students, long COVID, menstruation, SARS-CoV-2, sequelae

## Abstract

This study aimed to explore the potential application value of acupuncture in alleviating the impact of long COVID on women’s menstrual cycles, by investigating the occurrence of long COVID among female college students, its effects on menstruation, and the intervention of acupuncture. This cross-sectional study surveyed female college students with a history of coronavirus disease 2019 (COVID-19) before April 10, 2023. A questionnaire was used to analyze demographic characteristics, post-COVID sequelaes, duration of symptoms, and treatments received during that period. Among the 731 participants enrolled in the survey, 468 were female undergraduate students who met the analysis criteria. Among them, 85 individuals fit the definition of “Long COVID” (18.16%). Within the group of patients with long COVID, 69 individuals experienced changes in their overall menstrual patterns compared to the 6 months prior to contracting the novel coronavirus (81.18%). Additionally, 17 individuals opted for acupuncture treatment following the onset of COVID-19 (20.00%), which resulted in less impact on their menstrual cycle (41.18% vs 64.71% without receiving acupuncture, OR = 2.62), menstrual period duration (41.18% vs 64.71%, OR = 2.62), menstrual flow (47.06% vs 69.18%, OR = 2.52), and the color of menstrual blood (41.18% vs 63.24%, OR = 2.46) among these patients. Long COVID had a certain impact on menstruation. Acupuncture potentially alleviates the clinical symptoms of long COVID and reduces its impact on women’s menstrual cycle, thus having potential therapeutic value in the treatment of long COVID.

## 1. Introduction

Patients with coronavirus disease 2019 (COVID-19) often experience prolonged postillness symptoms. In light of this, the World Health Organization has proposed the definition of “Long COVID,”^[1]^ referring to individuals who may have or have been diagnosed with COVID-19 and continue to experience symptoms for at least 2 months, persisting beyond 3 months without other obvious triggers. These persistent long-term symptoms are known as COVID-19 sequelae. Approximately 10% to 20% of COVID-19 patients may contract “long COVID,” characterized by fatigue, cognitive impairment, muscular pain, and diminished olfactory or gustatory senses.^[2]^

Furthermore, SARS-CoV-2 has a certain impact on the reproductive system, particularly evident in its effects on menstrual health in women.^[3]^ Women are considered to be at high risk of contracting long COVID.^[4–7]^ However, there are currently no studies or reports available on the occurrence rate of long COVID and its impact on menstruation, specifically among young women with COVID-19. The function of menstruation encompasses several significant aspects in the female physiology. The normal functioning of the menstrual cycle ensures women have the opportunity to conceive and bear offspring. The different phases of the menstrual cycle are accompanied by fluctuations in estrogen and progesterone levels. The cyclical changes of menstruation help preserve a balance of these hormones, thereby sustaining the normal functioning of the female body. Furthermore, the female immune response is modulated by the hormones governing the menstrual cycle.^[8]^

To address this gap, this study focused on investigating female college students as the target population, considering the relatively controlled and less biased environment of university students. This study aimed to examine post-COVID-19 sequelae, duration of symptoms, treatments received, and changes in menstrual patterns compared with pre-COVID-19 conditions. The findings will provide evidence-based support for evaluating the impact of long COVID on reproductive health, especially menstrual-related issues, and the potential therapeutic role of nonpharmacological interventions in traditional Chinese medicine (TCM).

The nonpharmacological approach of TCM, especially acupuncture, has been proven to be effective in treating menstrual-related disorders. For instance, dysmenorrhea can be alleviated by acupuncture at Sanyinjiao (SP6) and Xuanzhong (GB 39).^[9]^ Menstrual disorder, oligo, or amenorrhea in women can be treated through acupuncture at Zhongji (CV 3), Qihai (CV 6), Guilai (ST 29), Sanyinjiao (SP 6), Yinlingquan (SP 9), Hegu (LI 4), and Neiguan (PC 6).^[10]^ Needling of specific acupuncture points such as Sanyinjiao (SP 6) may alter blood flow to the uterus and modulate prostaglandin levels.^[11]^ Furthermore, acupuncture can alleviate premenstrual syndrome.

In light of the existing research findings, this study aimed to explore the application value of acupuncture in alleviating the impact of long COVID on menstrual health, offering potential treatment options for young women seeking relief from both postillness symptoms and the effects of long COVID on reproductive health.

## 2. Data and methods

### 2.1. Survey objective

This study employed a cross-sectional design and used the QuestionStar platform to create a survey questionnaire. Snowball sampling was used to distribute the questionnaire via social media platforms to college students in multiple provinces and cities from March 10, 2023, to April 10, 2023. Participation in the survey was voluntary.

### 2.2. Eligibility criteria

The patients must have a confirmed medical history of COVID-19. Meet the World Health Organization’s definition of “long COVID”^[1]^: patients who may or have been diagnosed with COVID-19 and continue to experience symptoms for at least 2 months, persisting beyond 3 months without any other apparent cause; female students, with the age of 18 years and above, currently enrolled in higher education institutions. No history of ovarian dysfunction, endometrial disorders, uterine tumors, or menstrual cycle abnormalities within 6 months prior to the onset of COVID-19.

### 2.3. Survey methods

The snowball method was employed to share the questionnaire with students from various universities in different provinces and cities to improve the response rate. Logical conflicts in the questionnaire contents were identified and eliminated by the researchers to ensure data reliability. The study included voluntary participation of female college students with a history of COVID-19. They completed a self-designed “Post-COVID-19 Sequelae Survey” questionnaire, which collected information on age, menstrual status, clinical symptoms, and treatments received. The questionnaire covered general information about demographic characteristics, clinical symptoms, menstrual status, and treatments received. This included age, education level, age at menarche, medical history, long-term medication use, menstrual status within 6 months before and after the onset of COVID-19, and treatments received. Menstrual status assessment included menstrual cycle length, duration of menstruation, blood flow volume, blood color, presence of blood clots, premenstrual symptoms, date of the last menstrual period before COVID-19 onset, and dates of menstruation after COVID-19 onset. A change in menstrual cycle length of 3 or more days, compared to the 6 months preceding COVID-19 onset, was defined as a menstrual cycle alteration. The Medical Ethics Committee of Beijing University of Chinese Medicine Affiliated Dongzhimen Hospital approved the survey.

### 2.4. Sample size

A total of 731 questionnaires were collected. A total of 583 female participants were included in the survey, excluding 83 invalid participants, resulting in 500 valid responses and an effective response rate of 85.76%. Five individuals were excluded because they were under the age of 18 years, whereas 27 individuals were excluded due to menstrual cycle abnormalities within the 6 months prior to contracting COVID-19.

### 2.5. Statistical analyses

Statistical analyses were performed using SPSS version 25.0. Descriptive statistics were presented as mean ± standard deviation (*x̄* ± *s*). Categorical variables and ordinal variables were described as *N* (%). In this study, the menstrual cycle, menstruation duration, and intensity of dysmenorrhea were found to have non-normal distributions. Therefore, the Wilcoxon signed-rank test were used for analysis. Inter-group data comparisons were conducted using odds ratios. All statistical tests were 2-tailed, and statistically significant was set at *P* < .05.

## 3. Result

### 3.1. Demographic characteristics and clinical symptoms of “long COVID” patients

**Figure 1.**
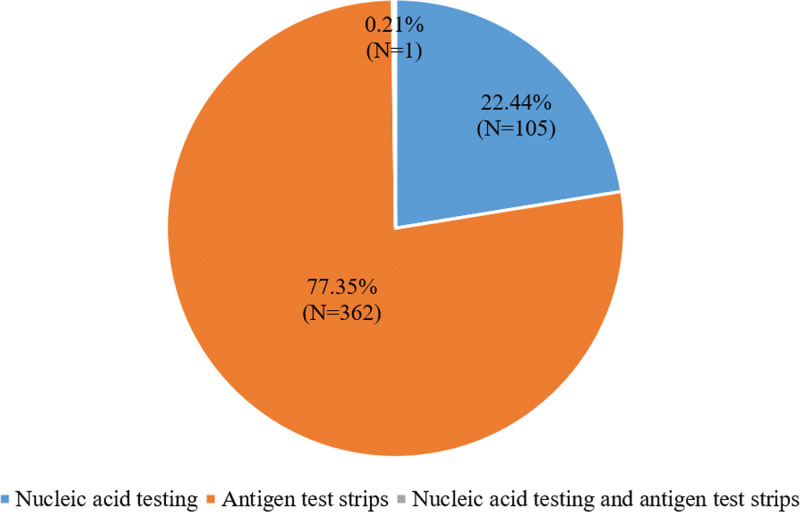
The method for confirming a diagnosis of COVID-19.

This study included 468 female university students with a history of COVID-19. Their ages ranged from 18 to 32 years old. The patients were diagnosed with COVID-19 through nucleic acid testing or antigen test strips (Fig. 1). Among all patients, 18.16% met the criteria for “ Long COVID” (*N* = 85) (Table 1).

**Table 1:** Demographic characteristics of “long COVID” patients.

Demographic characteristics (*N* = 85)	*N* (%)
Age	
18–22 yr	42 (49.41%)
23–27 yr	32 (37.65%)
28–32 yr	11 (12.94%)
Age at menarche	
10–11 yr	10 (11.76%)
12–13 yr	34 (40.00%)
14–15 yr	30 (35.29%)
≥16 yr	11 (12.94%)
Education	
Junior college students	1 (1.18%)
Undergraduate students	41 (48.24%)
Master degree candidate	32 (37.65%)
Doctoral candidate	11 (12.94%)
History of COVID-19 vaccination	
None	4 (4.71%)
Received 1 dose	4 (4.71%)
Received 2 doses	16 (18.82%)
Received 3 doses	61 (71.76%)
Medical history	
Respiratory system diseases	2 (2.35%)
Surgical diseases	1 (1.18%)
Other medical history, unspecified	2 (2.35%)
None	79 (92.94%)

The primary clinical symptoms observed in “long COVID” patients include fatigue (50.59%), cough (47.06%), dyspnea (23.53%), and expectoration (21.18%). A minority of the patients also experienced anxiety (12.94%), myalgia (11.76%), insomnia (12.94%), excessive sleepiness (2.35%), sore throat (1.18%), cognitive fog (1.18%), palpitations (1.18%), and various other symptoms (5.88%).

### 3.2. Menstrual status of “long COVID” patients

This survey revealed that 81.18% of patients with long COVID experienced changes in their overall menstrual status compared to 6 months prior to COVID-19 infection (*N* = 69).

Among the patients, 60.00% experienced changes in their menstrual cycle (*N* = 51). 22.35% reported a shortened cycle (*N* = 19), with an average reduction of (5.11 ± 2.28) days, whereas 37.65% reported a prolonged cycle (*N* = 32), with an average extension of (5.81 ± 2.19) days. Before the onset of COVID-19, the average menstrual cycle of the patients was (28.88 ± 3.27) days, which increased to (29.96 ± 5.73) days after contracting long COVID. This difference was statistically significant (*P* = .011 < .05).

Furthermore, 65.88% of patients experienced changes menstruation duration (*N* = 56). About 56.47% reported a prolonged menstruation period (*N* = 48), with an average reduction of (2.54 ± 1.37) days, whereas 9.41% reported a prolonged menstruation period (*N* = 8), with an average extension of (3.50 ± 1.85) days. Prior to COVID-19 infection, the average duration of menstruation for the patients was (5.94 ± 1.76) days, which increased to (7.71 ± 2.41) days after contracting long COVID. This difference was statistically significant (*P* < .001).

Additionally, 65.88% of the patients experienced changes in menstrual blood flow (*N* = 56) (Table 2).

**Table 2:** Menstrual blood flow in “long COVID” patients.

Menstrual blood flow (*N* = 85)	*N* (%)
Before COVID-19 infection	
Normal (≥20 mL and ≤80 mL)	52 (61.18%)
Hypomenorrhea (<20 mL)	26 (30.52%)
Hypermenorrhea (>80 mL)	7 (8.24%)
After contracting “long COVID”	
Decreased	42 (49.41%)
Increased	13 (15.29%)

**Figure 2.**
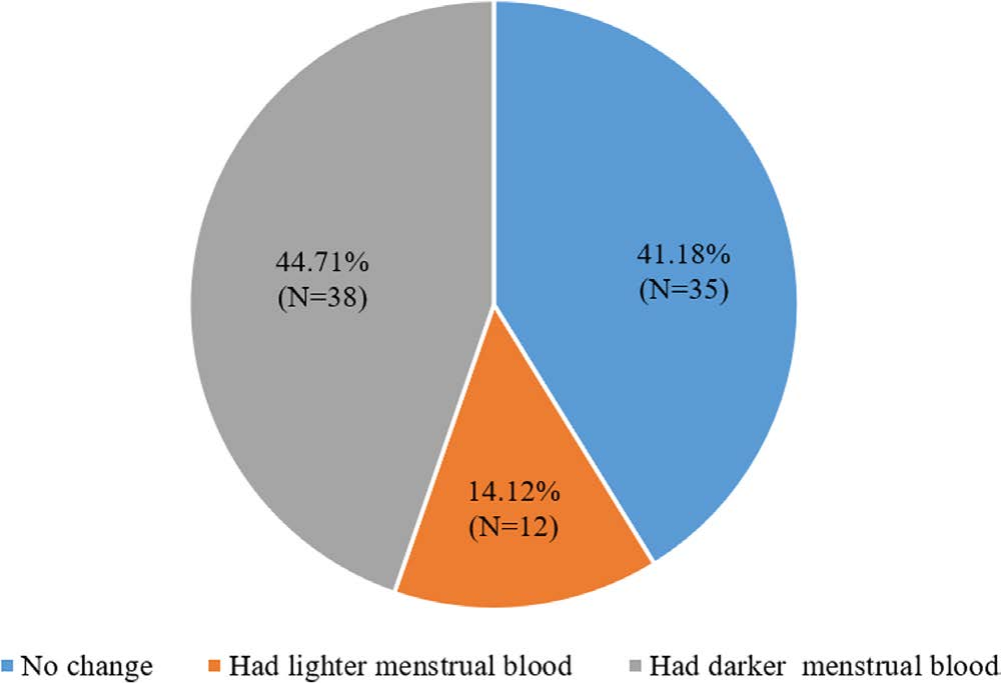
The changes in patients’ menstrual blood color.

Prior to COVID-19 infection, 8.24% of the patients had pale red menstrual blood (*N* = 7), 62.35% had bright red menstrual blood (*N* = 53), and 29.41% had dark red menstrual blood (*N* = 25). The changes in their menstrual blood color are as follows (Fig. 2).

Furthermore, 89.41% of the patients observed changes in the presence of blood clots during menstruation (*N* = 76) (Table 3).

**Table 3:** Presence of blood clots during menstruation in “long COVID” patients.

Characteristics of menstrual blood clots (*N* = 85)	*N* (%)
Before COVID-19 infection	
Menstrual blood clot volume	
None	27 (31.76%)
Small amount	45 (52.94%)
Large amount	13 (15.29%)
Menstrual blood clot color	
Pale red	4 (4.71%)
Bright red	28 (32.94%)
Dark red	26 (30.59%)
After contracting “long COVID”	
Menstrual blood clot volume	
Decreased	13 (15.29%)
Increased	45 (52.94%)
Menstrual blood clot color	
Lighter	15 (17.65%)
Darker	22 (25.88%)

The severity of menstrual pain in patients was assessed using the visual analog scale scoring method, which categorizes menstrual pain into 5 levels: no pain, mild pain, tolerable pain, severe pain, and excruciating pain. Changes in the severity of menstrual pain were observed in 32.94% of patients (*N* = 28). Among them, 8.24% reported an increase in pain (*N* = 24), while 4.71% reported a decrease in pain (*N* = 4) (Table 4). Additionally, it was observed that the severity of menstrual pain generally worsened after contracting long COVID (*U*_post-infection_ > *U*_pre-infection_, *P* < .001).

**Table 4:** Severity of menstrual pain in “long COVID” patients.

Level of dysmenorrhea (*N* = 85)	Preinfection, *N* (%)	Postinfection, *N* (%)
Absence of pain	64 (75.29%)	51 (60.00%)
Slight discomfort	9 (10.59%)	4 (4.71%)
Tolerable pain	9 (10.59%)	20 (23.53%)
Severe pain	2 (2.35%)	8 (9.41%)
Excruciating agony	1 (1.18%)	2 (2.35%)

About 55.29% of the patients newly contracted premenstrual symptoms (*N* = 47), with the main symptoms were breast tenderness (11.76%), lower back pain (16.47%), chills (14.12%), headache (14.12%), irritability (9.41%), abdominal diarrhea (4.71%), and excessive sleepiness (1.18%).

### 3.3. Treatment received by “long COVID” patients

A total of 95.29% of long COVID patients received appropriate treatment after contracting COVID-19 (*N* = 81). A total of 60.00% underwent Western medicine treatment (*N* = 51), 57.65% received TCM treatment (*N* = 49), and 22.35% received nonpharmacological treatment from TCM (*N* = 47), such as acupuncture (20.00%), massage therapy (4.71%), and cupping (1.18%).

**Figure 3.**
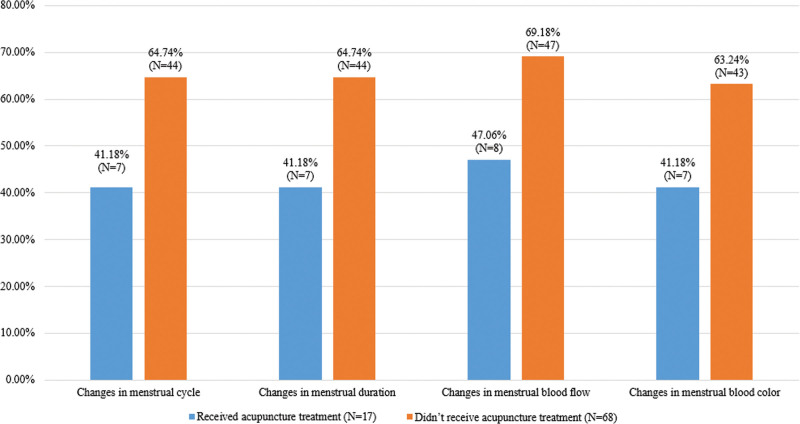
Comparison of menstrual Changes in “long COVID” patients who received acupuncture treatment and those who did not receive.

Among the long COVID patients surveyed in this study, the menstrual changes of those who received acupuncture treatment (*N* = 17) differed from those who did not receive acupuncture treatment (*N* = 68) (Fig. 3, Table 5).

**Table 5:** Comparison of menstrual changes in “long COVID” patients who received acupuncture treatment and those who did not receive.

Menstrual characteristics	Received acupuncture treatment (*N* = 17)	Did not receive acupuncture treatment (*N* = 68)	Odd ratio (OR)
Changes in menstrual cycle	7 (41.18%)	44 (64.71%)	2.62
Changes in menstrual duration	7 (41.18%)	44 (64.71%)	2.62
Changes in menstrual blood flow	8 (47.06%)	47 (69.18%)	2.52
Changes in menstrual blood color	7 (41.18%)	43 (63.24%)	2.46

## 4. Disscusion

### 4.1. The impact of long COVID on menstruation

Previous studies have found that various viruses, such as human immunodeficiency virus, human papillomavirus (HPV), and hepatitis virus, have a certain impact on women’s reproductive health related to menstruation. Research has confirmed^[12,13]^ that the influence of SARS-CoV-2 on the female reproductive system is related to the angiotensin-converting enzyme^[2]^ (ACE2) receptor, which is expressed in the ovaries, fallopian tubes, uterus, cervix, and vagina. ACE2 is involved in regulating corpus luteum angiogenesis and deformation, while also affecting regular changes in endometrial tissue.^[13]^ ACE2 is relatively highly expressed in ovaries and oocytes,^[14]^ and the invasion of SARS-CoV-2 into host cells requires the combined action of transmembrane serine protease 2 (TMPRSS2) and ACE2. Therefore, ovaries and oocytes may be potential targets of SARS-CoV-2’s impact on the female reproductive system. The development of patient follicles and maturation of oocytes are affected by the virus, resulting in disrupted ovulation cycles and changes in menstrual cycle duration. Research has found^[11]^ that there is no significant overall change in estrogen levels in reproductive-aged women with COVID-19; however, some patients have abnormally elevated levels of follicle-stimulating hormone and luteinizing hormone, which is inconsistent with their age and anti-Müllerian hormone levels, suggesting inhibited ovarian function. However, no changes in ovarian reserve function were observed, indicating that inhibition of ovarian function may be the cause of changes in menstrual flow and blood clotting patterns. Additionally, SARS-CoV-2 can alter the function of the hypothalamic–pituitary–gonadal axis, thereby reducing estrogen and progesterone synthesis.^[15]^ There is currently limited research on the effects of long COVID on the endometrium and ovarian function in women, and the mechanisms by which it affects female reproductive function related to menstruation remain unclear and require further study.

In addition, long COVID can cause persistent immune inflammation in the body, leading to organ dysfunction.^[16,17]^ In this process, interleukin-1β (IL-1β) plays an important role as a pro-inflammatory cytokine receptor agonist.^[18]^ IL-1β can activate the inflammatory process, stimulate the metabolism of arachidonic acid in the endometrial villi, and consequently cause an increase in prostaglandins,^[19]^ leading to dysmenorrhea. These symptoms are also influenced by patients’ mental and psychological state. Studies have shown^[20]^ that premenstrual or menstrual symptoms can have a significant impact on women’s health and are closely related to daily life and psychological disorders. During the course of long COVID, patients experience significant stress, often accompanied by anxiety, depression, or sleep disorders, resulting in psychological tension that may worsen dysmenorrhea or other premenstrual symptoms.

### 4.2. The intervention effect of acupuncture on long COVID

In this study, only 22.35% of patients with long COVID received nonpharmacological treatment in TCM, and 20.00% received acupuncture. This may be attributed to the rigid perception that acupuncture is merely a preventive measure that cannot treat infectious diseases. However, acupuncture has a history of thousands of years in preventing and treating infectious diseases, with the earliest records found in *Huangdi Neijing*. The principle of acupuncture treatment as stated in the *Suwen* section, emphasizes the need to address stagnation, nourish vitality, strengthen weakness, reduce excess, and alleviate suffering.

Acupuncture has been shown to improve cardiovascular dysfunction, respiratory symptoms, neurological symptoms, digestive system symptoms, endocrine symptoms, and other manifestations in patients with long COVID.^[2]^ The effect of acupuncture on COVID-19 is multi-targeted,^[21]^ involving the production of 2 active compounds and the modulation of 180 protein targets. These effects can be summarized as improving lung function, regulating innate immunity, balancing antiinflammatory and pro-inflammatory factors, and activating the vagus nerve-cholinergic pathway through the activation of neuroactive ligand-receptor interactions, cancer pathways, and related signaling pathways, to protect the lungs. Existing research^[22]^ has confirmed that acupuncture can promote humoral and cellular immunity, enhance natural killer cell activity, and activate the neuro-endocrine-immune response. This neuro-inflammatory pathway regulates inflammatory factors more rapidly and specifically than the humoral antiinflammatory pathway does. Patients with long COVID often experience increased reactive oxygen species (ROS) in their bodies, leading to tissue damage and reduced levels of nitric oxide (NO).^[23]^ Acupuncture can elevate NO levels, promote local circulation, inhibit viral replication, and enhance immune responses.^[24]^ Additionally, acupuncture can also regulate ROS, reduce oxidative stress, and suppress inflammation, thereby alleviating clinical symptoms among long COVID patients.^[25,26]^

### 4.3. Alleviating the impact of long COVID on menstruation through acupuncture

The likelihood of females contracting a long COVID is 4 times higher than that of males.^[27]^ Furthermore, SARS-CoV-2 has certain effects on the female reproductive system. Therefore, the search for treatment methods that can both relieve post-COVID sequelae and mitigate the reproductive health impact of long COVID in females remains an urgent clinical concern. This study observed that among patients with long COVID who received nonpharmacological TCM treatment, the number of individuals experiencing disruptions in menstrual cycle, duration, blood volume, and color was significantly lower than those who did not receive such treatment, with statistically significant differences.

In clinical practice, hormone therapy is commonly used to treat menstruation-related disorders in women. In recent years, acupuncture have been gradually employed in the treatment of menstrual disorders. One study implemented a staged treatment approach for menstrual irregularities,^[28]^ targeting acupoints such as Xuehai (SP10), Sanyinjiao (SP6), Taixi (KI3), and Zhaohai (KI6) during the follicular phase, along with respiratory regulation techniques. During the ovulatory phase acupoints including Ganshu (BL18), Sanyinjiao (SP6), Hegu (LI4), Taichong (LR3), Tianshu (ST25), Zhongji (CV3), and Zigong (EX-CA1), were selected to promote the discharge of ova. Patients treated according to this protocol achieved normalized menstrual cycles without recurrence during the 6-month follow-up period. Additionally, studies have shown that acupuncture has favorable therapeutic effects on dysmenorrhea,^[29,30]^ effectively reducing pain intensity, muscle spasms, and various systemic symptoms.

TCM holds the belief that “the kidneys are the foundation of congenital essence,” and Tian Gui (the essence related to menstruation) is produced by the kidneys. The *Shang Gu Tian Zhen Lun of Su Wen* states, “When Tian Gui arrives, the Ren meridian is unobstructed, the Tai meridian and Chong meridian are abundant, and the women commence experiencing menstruation, thus enabling pregnancy.” It further explains, “When kidney’s qi is abundant and Tian Gui arrives, the essence overflows, yin and yang harmonize, and pregnancy can be achieved.” The *Fu Qing Zhu Nv Ke* records, “Menstruation originates from the kidneys. In *Volume 153 of Sheng Ji Zong Lu*, it is mentioned that the lack of pregnancy in women is often due to insufficient Chong meridian and Ren meridian and deficient kidney’s qi with coldness. Therefore, it is evident that female menstruation is closely related to kidney qi, the Chong and Ren meridians, and the uterus. Research has found a high similarity between the kidney-Tian Gui-the Chong and Ren meridians-uterus axis and the hypothalamic–pituitary–ovarian axis.^[31]^ For patients with long COVID, long-term illness leads to depletion of righteous qi, deficient blood supply to the kidneys, insufficient the Chong and Ren meridians, and menstrual disturbances. By regulating the meridians, promoting circulation of qi and blood, and nourishing kidney qi, acupuncture aims to restore harmonious the Chong and Ren meridians and achieve a state of yin–yang balance to restore the functionality of the uterus and achieve therapeutic goals.

From a treatment mechanism perspective, acupuncture can exert its effects by regulating the levels of progesterone, estrogen, and prostaglandins, promoting follicular development, improving endometrial thickness, and modulating neuro-immune pathways. Studies have found that acupuncture at the Neiguan (PC6), Guanyuan (CV4), Zusanli (ST36), and Sanyinjiao (SP6) acupoints can indirectly regulate the hypothalamic–pituitary–ovarian axis, decrease androgen levels, and promote follicle growth.^[32]^ Additionally, acupuncture can regulate uterine microcirculation, enhance the expression of estrogen and progesterone receptors on the surface of the endometrium, and promote endometrial regeneration.^[33,34]^ Studies have also revealed that acupuncture at Guanyuan (CV4) and Zigong (EX-CA1) can regulate uterine smooth muscle electrical activity and the neuro-endocrine system, thereby increasing uterine blood flow.^[35,36]^ Moreover, patients with menstrual disorders often exhibit elevated levels of inflammatory cytokines in the ovarian tissues, endometrial cells, and follicular fluid.^[37]^ Research has shown that acupuncture can improve metabolic function and blood circulation in the rat uteri, enhance tissue nutrition, and alleviate local inflammation.^[38]^ Furthermore, acupuncture has dual immune regulation and antiinflammatory effects. It promotes macrophage polarization and regulates pro-inflammatory cytokines such as tumor necrosis factor-α, IL-12, IL-23, IL-6, and IL-1β.^[39]^ Acupuncture not only improves menstrual disorders but also alleviates immune inflammation caused by long COVID, mitigates symptoms, and reduces prostaglandin levels, thus relieving dysmenorrhea.

The study revealed that patients with long COVID who received nonpharmacological treatment in acupuncture demonstrated a significantly lower incidence of menstrual changes compared to individuals who did not participate in acupuncture interventions. However, the study still had potential limitations: 68 subjects may have received some other treatment but they did not receive acupuncture treatment, it can be massage cupping, western or actually combined. For acupuncture, location, dosage and methods were not identified.

## 5. Conclusion

Long COVID has a discernible impact on the reproductive health of women, as evidenced by this study. Among female college students who contracted long COVID, a notable number experienced changes in their menstrual patterns. These alterations encompassed variations in menstrual cycle duration, length of menstruation, volume and color of menstrual blood, presence of blood clots, and modifications in premenstrual symptoms. Additionally, the study revealed a statistically significant disparity: patients with long COVID who received nonpharmacological treatment in acupuncture demonstrated a significantly lower incidence of menstrual changes compared to individuals who did not participate in acupuncture interventions.

Currently, the treatment options for long COVID remain limited. Acupuncture, a time-honored nonpharmacological therapeutic approach for TCM, has emerged as a viable solution. By alleviating the clinical manifestations associated with long COVID, acupuncture effectively mitigates its repercussions on women’s menstrual health. Consequently, acupuncture has a certain practical value in the treatment of long COVID.

## Author contributions

**Writing—original draft:** Juwei Dong.

**Writing—review & editing:** Jinxia Ni, Ziniu Zhang.

**Investigation:** Haoyue Yan, Jingni Xu, Jingjing Zhao.
